# Forces and energetics of the canonical tetrameric cation channel gating

**DOI:** 10.1073/pnas.2221616120

**Published:** 2023-07-03

**Authors:** Simon Scheuring

**Affiliations:** ^a^Department of Anesthesiology, Weill Cornell Medicine, New York, NY 10065; ^b^Department of Physiology and Biophysics, Weill Cornell Medicine, New York, NY 10065; ^c^Kavli Institute at Cornell for Nanoscale Science, Cornell University, Ithaca, NY 14853

**Keywords:** ion channel, K^+^-channel, forces, single molecule biophysics, polymer extension

## Abstract

The canonical gating mechanism of tetrameric cation channels involves the spreading of the pore-lining helices at the so-called bundle-crossing gate. Despite a wealth of structural information, we lack a physical description of the gating process. Here, I took advantage of an entropic polymer stretching physical model and MthK structures to derive the forces and energies involved in pore-domain gating. In MthK, the Ca^2+^-induced conformational change in the RCK domain alone opens the bundle-crossing gate through pulling via unfolded linkers. In the open conformation, the linkers serve as entropic springs between the RCK domain and bundle-crossing gate that store an elastic potential energy of 3.6*k*_B_T and exert 9.8 pN (piconewton) radial pulling force to keep the gate open. I further derive that the work to load the linkers to prime the channel for opening is up to 3.8*k*_B_T, exerting up to 15.5 pN to pull the bundle-crossing open. Opening of the bundle-crossing leads to a release of 3.3*k*_B_T spring potential energy. Thus, the closed/RCK-apo and the open/RCK-Ca^2+^ conformations are separated by a barrier of several *k*_B_T. I discuss how these findings relate to the functional properties of MthK and suggest that given the architectural conservation of the helix–pore-loop–helix pore-domain among all tetrameric cation channels, these physical parameters might be quite general.

## Significance

All tetrameric cation channels share the same pore domain with the characteristic helix–pore-loop–helix architecture. Canonical cation channel function involves ion selectivity by the pore loops and gating by movements of the pore-lining helices. However, much less is known about the physics of the gating process. Here, I took advantage of MthK structures and an entropic polymer stretching physics model to derive the forces and energies involved in channel gating. In MthK, conformational changes in the gating ring alone pull the channel open via unfolded linkers, offering the unique opportunity to use a physical model to calculate the forces, 9.8 pN (piconewton), and energies, 3.6*k*_B_T, involved in gating.

The K^+^ channel of streptomyces A (KcsA) X-ray structure unlocked ion channel structural biology (1). KcsA is a minimal canonical tetrameric cation channel pore domain, where each subunit comprises two transmembrane helices and a membrane-entrant extracellular pore loop. The four pore-loop copies form the potassium (K^+^) ion selectivity filter, while the pore-lining helices transmembrane helix 2 (TM2) (S6 in channels that comprise an S1 to S4 voltage sensor domain) form a closed bundle-crossing gate at the intracellular face. Methanobacterium thermoautotrophicum K^+^-channel (MthK) is a K^+^ channel that alike KcsA forms the canonical helix–pore-loop–helix pore domain without any other transmembrane domains, but also comprises an intracellular Ca^2+^-binding domain, the regulator of conductance of K+ (RCK), or gating ring. Accordingly, the X-ray structure of MthK in the presence of Ca^2+^, the second K^+^ channel structure solved, revealed a Ca^2+^-bound RCK ring and a pore domain with a wide-open intracellular bundle-crossing gate (2). Comparison of the KcsA and MthK structures established the canonical model of tetrameric ion channel structure and function, where the pore loop forms the ion selectivity filter with the characteristic conserved TVGYGD sequence (for K^+^ selectivity) and the bundle crossing forms the conductance gate that is modulated by an equally conserved Gly hinge in the pore-lining helix (3). Interestingly, in the MthK crystal structure, the residues between the RCK ring and pore domain, though essential for transmitting the information of the conformational state of the RCK ring to the pore domain, remained unstructured and unresolved (2).

All tetrameric K^+^-, Na^+^-, and Ca^2+^ channels, also those from families that feature additional ligand-binding or sensory (e.g., voltage-sensor or voltage-sensor-like) domains, e.g., K_ir_, K_Ca_, K_V_, K_2P_, CNG, HCN, Ca_V_, Na_V_, Transient receptor potential (TRP) channel, polycystic (P) family (TRPP), mucolipin (TRPML), vanilloid (TRPV), canonical (TRPC), and melastatin (TRPM), share the canonical pore-domain architecture, with the characteristic “helix–pore-loop–helix” structure (4). A plethora of structures from all families revealed the canonical pore-domain architecture, and some degree of bundle-crossing movements is observed in all cases where closed and open channel structures are available. Even channels (e.g., TREK-2 in the K_2P_ family) that gate at the selectivity filter couple pore-helix movements at the bundle-crossing to gate the filter (5). Thus, I propose, and think it is a rather accepted view, that a radial outward pulling force (with respect to the fourfold axis) on the pore-lining helices at the intracellular bundle crossing is an essential and general process in gating.

**Fig. 1.**
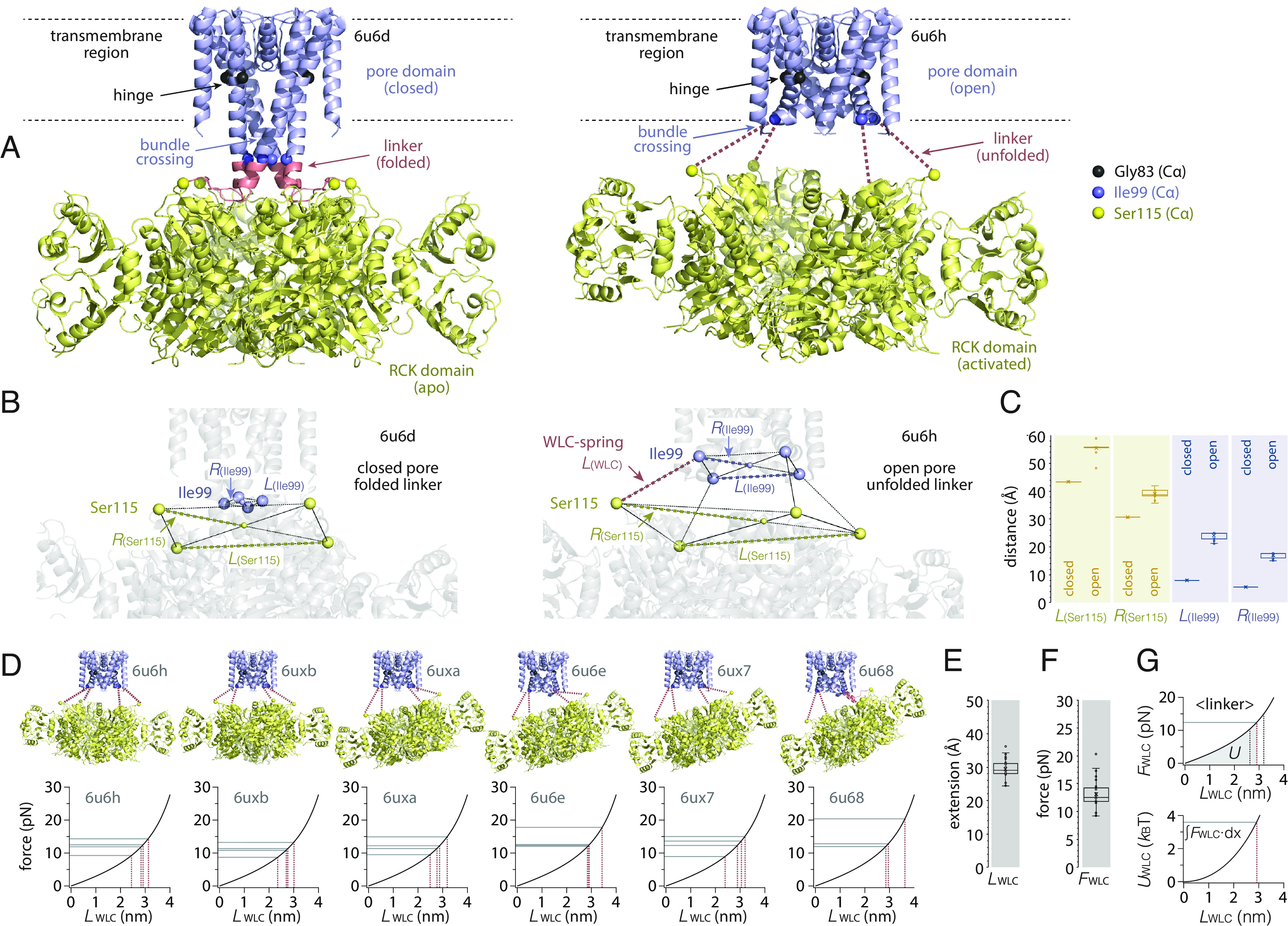
Worm-like chain (WLC) springs connect the RCK ring to the pore-domain bundle-crossing gate in MthK open conformation structures. (*A*) Cryo-EM structures (PDB accession codes indicated) of the closed (*Left*) and an open (*Right*) conformation. The pore domain is shown in blue (residues 18 to 99), the linker is shown in red (residues 100 to 114), and the RCK domain is shown in yellow (residues 115 to 338). While the linker is unfolded (and unresolved) in the open state (*Right*, red dashed lines), it is folded and resolved in the closed state (*Left*). The closed-state folded linker extends the pore-lining helix (residues 100 to 105) and is ordered and associated with the RCK domain but without secondary structure (residues 106 to 114). The Cα of the key residues, Gly83 (glycine hinge, black), Ile99 (last resolved residue on the pore domain in the open state, blue), and Ser115 (first resolved residue on the RCK domain in the open state, yellow) are shown as spheres. (*B*) Close-up view of the linker region in the closed (*Left*) and an open (*Right*) conformation: The Cα atoms of Ser115 and Ile99 are shown as yellow and blue spheres, respectively, while the structures are shown in transparent gray for clarity. In the open conformation, Ser115 and Ile99 are connected by a 16-residue long unfolded linker polypeptide that can be modeled as a worm-like-chain spring (WLC spring, red dashed line). (*C*) Distance analysis of Ser115 and Ile99 Cα atoms. Ser115 (yellow): Upon Ca^2+^ binding to the RCK ring, Ser115 Cα spread interresidue distances, *L*_(Ser115)_, from 43.4 ± 0.0 Å (*n* = 4) to 55.8 ± 0.8 Å (*n* = 24) (yellow dashed lines *L*_(Ser115)_ in *B*), and radius, *R*_(Ser115)_, from 30.7 ± 0.0 Å (*n* = 4) to 39.3 ± 1.3 Å (*n* = 24) (yellow dashed lines *R*_(Ser115)_ in *B*). Ile99 (blue): Upon bundle-crossing opening, Ile99 Cα spread interresidue distances, *L*_(Ile99)_, from 7.9 ± 0.0 Å (*n* = 4) to 24.0 ± 1.3 Å (*n* = 24) (blue dashed lines *L*_(Ile99)_ in *B*), and radius *R*_(Ile99)_, from 5.6 ± 0.0 Å (*n* = 4) to 17.0 ± 1.0 Å (*n* = 24) (blue dashed lines *R*_(Ile99)_ in *B*). (*D*, *Top*) Cryo-EM structures (PDB accession codes indicated) of the six open conformations. The linkers between Ser115 and Ile99 are unfolded and unresolved and indicated by red dashed lines. (*Bottom*) WLC force-extension graphs (black) with Ser115-to-Ile99 extension distances for each individual linker (red dashed lines) and corresponding forces (gray lines) for each open conformation structure (PDB accession codes indicated). (*E*) Ser115-to-Ile99 WLC-spring extension *L*_(WLC)_ = 29.4 ± 3.1 Å (*n* = 23), generates (*F*) a pulling force *F*_(WLC)_ = 13.1 ± 2.8 pN (*n*=23). (*G*) The integration of the WLC-spring extension (*Top*: gray shaded area labeled *U* for <*L*_WLC_>) provides the elastic WLC spring potential energy in the extended open-state linkers <*U*_WLC_> = 3.6 *k*_B_T (*Bottom*).

Considering proteins as nanoscopic physical machines, it is a timely and worthy challenge to decipher the physical properties of the action of biomolecules: In the case of canonical tetrameric cation channels, this is to elucidate the forces and energetics of bundle-crossing gating (Fig. 1*A*).

A dream experiment for single-molecule biophysicists would be to pull an ion channel open—using an AFM (atomic force microscopy) tip (6) or an optical tweezer (7) — and directly measure the forces and energies involved in the process. This has, to the best of my knowledge, remained impossible, and such experiments would not only be extremely difficult but might be flawed by imprecisions in the calibration of the system, in vectorial pulling, and difficulties in measuring low piconewton (pN) forces.

Here, I take advantage of a serendipitous molecular system to solve this problem. I determine the forces and energetics of tetrameric ion channel bundle-crossing gating in the *Methanobacterium thermoautotrophicum* K^+^ channel MthK. MthK is a purely Ca^2+^-gated channel with no additional voltage-sensing or allosteric domain (8). Also, it has open probabilities close to 0 in the absence and close to 1 in the presence of Ca^2+^ (2, 9–12). The first MthK X-ray structure readily revealed an open channel with a widely spread bundle-crossing gate and disordered linkers between the pore domain and RCK ring (2, 8). However, a recent cryogenic electron microscopy (cryo-EM) study provided also the closed conformation with a folded linker between the pore domain and the RCK-apo (Fig. 1 *A*, *Left*), and a wealth of open conformation MthK structures from channels in nanodisks tumbling freely in (vitrified) solution (13), i.e., not subjected to crystal packing constraints. The open-state MthK structures confirmed the open-pore conformation and that the Ca^2+^-bound RCK ring was separated from the pore domain through unfolded linkers (Fig. 1 *A*, *Right*). In addition, as the molecules were not constrained in a crystal, the study revealed that the Ca^2+^-bound RCK ring tumbled freely underneath the pore domain adopting a continuum of angular arrangements with respect to the pore (Fig. 1*D*). Taking advantage of these structures (13) and a polymer extension physical model, the worm-like chain (WLC) model (14), which allows to calculate the spring properties of unfolded polypeptide stretches such as the unfolded linker, I derive the forces and energetics of bundle-crossing gating.

## Results

### MthK, an Ideal Channel: Molecular Simplicity and a Wealth of Structural Information.

Recent advances in cryo-EM and image processing (15, 16) now allow solving membrane protein structures rather routinely (17). Importantly, the avoidance of crystallization and imaging of single particles in (vitrified) solution allows now to calculate a variety of representative structures of statistical ensembles (18, 19). In the case of the Ca^2+^-gated K^+^ channel MthK, Fan et al. (13) were able to report a closed channel in the absence of Ca^2+^ (Fig. 1 *A*, *Left*) and several open structures in the presence of Ca^2+^ (Fig. 1 *A*, *Right* and Fig. 1 *D*, *Top*). The Ca^2+^-bound activated RCK ring was observed in different tilted orientations with respect to the pore domain. Indeed, the authors commented that many more gating-ring tilts existed (13). Thus, cryo-EM and image processing were able to capture and computationally pool particles from a structural continuum of a channel with an activation domain freely floating underneath the pore domain. While the closed-state structure featured straight pore-lining helices and folded linkers between the pore domain and RCK ring (Fig. 1 *A*, *Left*), all open-state structures had a quasi-identical pore domain with kinked open pore-lining helices, unfolded linkers, and the floating RCK ring (Fig. 1 *A*, *Right* and Fig. 1 *D*, *Top*). The pore domain in all open conformation structures was also nearly identical to the former X-ray structure with the pore-lining helix being bent at the so-called hinge glycine, G83 (2, 8, 13). All 6 open conformation cryo-EM structures resolved the 4 subunits’ pore domains up to residue Ile99 and the RCK ring starting at Ser115 (except for one subunit; see *A Notable Outlier*, *Discussion*). Thus, the wealth of the cryo-EM structures allowed me to analyze the Cα coordinates of Ile99 and Ser115 and the variability of the relative orientation and extension of the 16-amino-acid-long unfolded linkers in 23 subunits (Fig. 1*B*). These analyses showed how the radial outward movement of Ser115 on the RCK ring (Fig. 1*B*, yellow spheres, Fig. 1*C*, yellow distributions) leads to an opening at the bundle-crossing gate represented by the outward movement of Ile99 (Fig. 1*B*, blue spheres, Fig. 1*C*, blue distributions). But by what mechanism and how much force is generated from the RCK ring to pull the bundle-crossing gate? This seems particularly enigmatic given that the linkers are all of variable orientation and length and are unfolded and unstructured.

### Entropic Spring, an Ideal Pulling Experiment: Well Calibrated, Soft, and at Equilibrium.

Single-molecule experiments have allowed biophysicists to quantitatively describe forces in molecular processes (7, 20). Typically, AFM (6, 7, 21) or optical tweezers (6, 7, 22) were used to manipulate biomolecules. Stretching polymers such as polyethylene glycol, DNA, RNA, and polypeptide chains (in protein unfolding experiments) using AFM and optical tweezers provided insights into polymer physics experimentally and were combined with and/or stimulated theory (14, 23) providing access to equilibrium energetics through the Jarzynski theorem (24). Such experiments showed that the force involved in stretching a polypeptide stretch was accurately described by the WLC model (6, 14, 23): According to the WLC model, as the two ends of an unfolded amino acid chain are separated and the polypeptide is stretched, the number of possible polymer configurations decreases dramatically. Thus, a relaxed coiled peptide is a high-entropy and low free-energy state, while an extended peptide is a low-entropy and high free-energy state. The stretching process along the extension reaction coordinate is thus reported by an increase in force, where the polypeptide chain acts as an entropic spring. The force generated by a peptide chain depends on the contour length, *L_C_* (the length along the polymer backbone), which is a function of the number of chain units, *x*, and their persistence length, *l_p_* (the maximum extension per unit). For amino acids, *l_p_* was determined to be 0.38 nm in peptide stretching experiments (*Methods*). Thus, knowing the length, 16 residues, and measuring the end-to-end extension, i.e., the Ile99-to-Ser115 distances as derived from the Cα atomic coordinates of Ile99 and Ser115 (Fig. 1 *B*, *Right*, blue to yellow spheres), of the linkers in each subunit in each structure, I could determine the pulling forces between Ile99 and Ser115 (Fig. 1 *D*, *Bottom* and Eq. **7**). The length of each unfolded linker (labeled WLC spring in Fig. 1 *B*, *Right*, red dashed lines in Fig. 1 *D*, *Top*) in each structure could be plotted and the force that they generated derived from the WLC-spring force-extension graphs (Fig. 1 *D*, *Bottom*). The average extension length of the linkers was 29.4 ± 3.1 Å (Fig. 1*E*), generating an average force of 13.1 ± 2.8 pN (Fig. 1*F*). From the force-extension graphs, the elastic energy can be calculated through integration (Fig. 1 *G*, *Top*), giving a WLC-spring potential energy of MthK open conformation linkers of 3.6 *k*BT (Fig. 1 *G*, *Bottom* and Eq. **8**). All these values concern individual linkers.

### Gating Forces and Energetics.

**Fig. 2.**
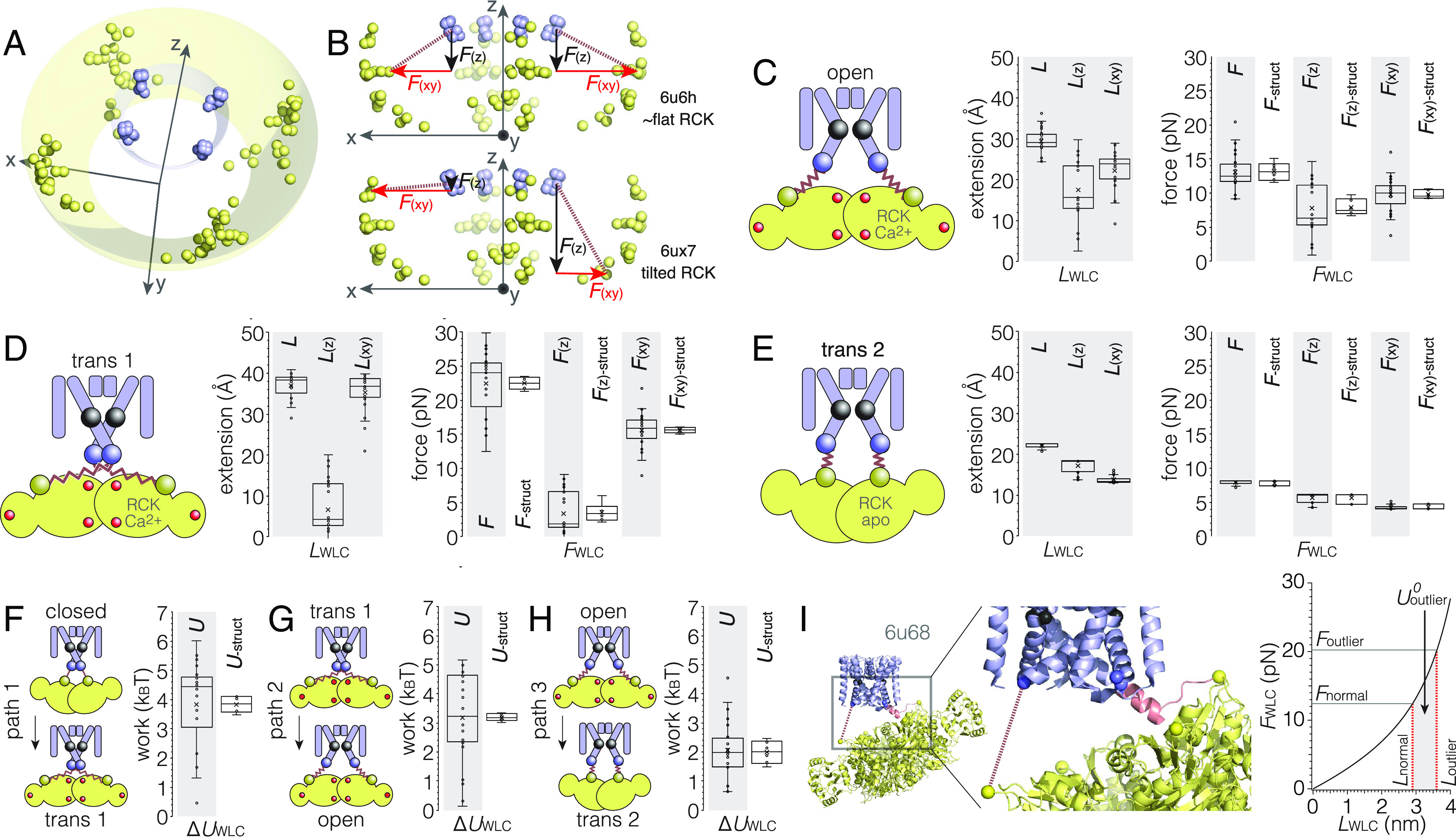
Forces between Ile99 at the bundle-crossing gate and Ser115 on the RCK ring in MthK. (*A*) and (*B*) 3D (*A*) and side (*B*) views of the Ser115 (yellow spheres) and Ile99 (blue spheres) Cα coordinates in all open-state structures. All structures were aligned to match the fourfold and pore axis with the z-coordinate axis. For clarity, all coordinates are displayed in a fourfold symmetrized way. (*A*) The Ser115 coordinates distribute roughly on a dome representing the space accessible by the floating RCK ring anchored to the pore domain over the four flexible linkers. (*B*) Side view of the coordinates to illustrate the positions of Ser115 pairs of oppositely located RCK subunits. Two examples are highlighted: In structure 6u6h (*Top*) that has an almost flat RCK ring, the two opposite Ser115 lie roughly in the plane and have similar z (black arrows, along the fourfold axis) and x, y (red arrows, mediating the radial outwards pull) components. In structure 6ux7 (*Bottom*) that has a highly tilted RCK ring, the two opposite Ser115 have very different z and x, y components, providing that the two linkers have very different vertical (black arrows) and radial (red arrows) pulling forces. The WLC springs (red dashed lines) thus generate forces with varying components *F*_(z)_ (black arrows) and *F*_(xy)_ (red arrows). (*C*–*E*, *Left*) Structural schematics. (*Center*) Total linker lengths, *L*, and length components in vertical, *L*_(z)_, and radial, *L*_(xy)_, directions. (*Right*) Total force, *F*, force components in vertical, *F*_(z)_, and radial, *F*_(xy)_, directions (*n* = 23, gray background), and average force values per structure, *F*_-struct_*F*_(z)-struct_, and *F*_(xy)-struct_ (*n* = 6, white background). (*C*) Open state: Ser115_(open)_ – linker WLC spring – Ile99_(open)_ (cryo-EM structures). The radial force, *F*_(xy)-struct_ = 9.9 ± 0.6 pN, keeps the bundle-crossing gate open. (*D*) Transition state 1: Ser115_(open)_ – linker WLC spring – Ile99_(closed)_. A high radial gating force, *F*_(xy)-struct_ = 15.6 ± 0.4 pN, pulls the bundle-crossing open. (*E*) Transition state 2: Ser115_(closed)_ – linker WLC spring – Ile99_(open)_. The radial pull, *F*_(xy)-struct_ = 4.2 ± 0.4 pN, between the apo-RCK and the open bundle crossing is low. Legend to schematics in *C*–*E*: Cα Gly83 (black sphere), Cα Ile99 (blue sphere), Cα Ser115 (yellow sphere), pore domain (blue), RCK domain (yellow), Ca^2+^ ions (red spheres), and unfolded WLC-spring linkers (red zigzag lines). (*F*) Path 1: Work performed moving Ser115_(closed)_ to Ser115_(open)_ between closed and transition 1 states, *U*_path 1_ = 3.8 ± 0.3 *k*_B_T. (*G*) Path 2: Work performed moving Ile99_(closed)_ to Ile99_(open)_ between transition 1 and open states, *U*_path 2_ = −3.3 ± 0.1 *k*_B_T. (*H*) Path 3: Work performed moving Ser115_(open)_ to Ser115_(closed)_ between open and transition 2 states, *U*_path 3_ = −1.8 ± 0.4 *k*_B_T (*I*) *A Notable Outlier*: In structure 6u68 (*Left*), one linker remained folded extending from the pore-lining helix setting the RCK domain in a strongly tilted arrangement, while the opposite linker is unfolded and in a highly stretched configuration (red dashed line) (*Middle*), allowing an estimate of the energy associated with linker folding as the potential energy difference between the WLC springs with extensions of the highly stretched linker (*L*outlier) and the mean of all canonical linkers (*L*normal), important to estimate channel closing in path 4 (*Discussion*).

Next, to extract the vectorial pulling forces on the bundle-crossing gate, I needed to calculate the parallelograms of force generated by each linker WLC spring. In the structures, the average linker between Ser115 and Ile99 pulls roughly with a 45° angle downward and radial outward with respect to the pore fourfold axis (Figs. 1 *B*, *Right*, 1*D* and 2 *A* and *B*). Since opening the bundle-crossing gate implies a radial-outward movement of the pore-lining helices, as reported by the Ile99 coordinates, below the hinge glycine, Gly83, I separated the WLC-spring forces into two orthogonal components, namely in the z-direction (coinciding with the fourfold and pore axis) (Fig. 2*B*, *F*_(z)_) and the x, y direction generating a radial outward pull from the pore axis (Fig. 2*B*, *F*_(xy)_). I hypothesize that only the component pulling radially in the x, y direction, *F*_(xy)_, generates the force to gate the bundle crossing (Fig. 2*B*). Thus, I calculated for all open-state structures (Fig. 2 *C*, *Left*) all linker lengths, *L*, and the vertical, *L*_(z)_, and radial, *L*_(xy),_ length components (Fig. 2 *C*, *Middle*), as well as the corresponding forces *F*, *F*_(z)_, and *F*_(xy)_ (Fig. 2 *C*, *Right*). I found linker WLC-spring pulling forces *F* = 13.1 ± 2.8 pN, *F*_(z)_ = 7.9 ± 3.7 pN, and *F*_(xy)_ = 9.8 ± 2.6 pN (Fig. 2 *C*, *Right*, gray shaded, *n* = 23).

An interesting aspect emerges from the tetrameric nature of the channels combined with the unstructured linkers and the floating RCK ring: Intuitively, two opposing subunits within the tetramer have approximately equal *F*_(z)_ and *F*_(xy)_ in molecules where the RCK domain is roughly flat with respect to the pore domain (Fig. 2 *B*, *Top*, 6u6h). In contrast, in molecules with a strongly tilted RCK ring, *F*_(z)_ and *F*_(xy)_ of opposing subunits diverge, with one having a large *F*_(z)_ and a small *F*_(xy)_ component and the other having a small *F*_(z)_ and a large *F*_(xy)_ component (Fig. 2 *B*, *Bottom*, 6ux7). However, because of the tetrameric nature of the channel and the resulting geometrical relationship of opposite-located subunits (Fig. 2 *A* and *B*), when I calculated the average force and average force components over the subunits for each structure, I received *F*_-struct_ = 13.2 ± 1.3 pN, *F*_(z)-struct_ = 8.0 ± 1.2 pN, and *F*_(xy)-struct_ = 9.9 ± 0.9 pN (Fig. 2 *C*, *Right*, white background, *n* = 6). Strikingly, the average forces among the different structures are highly similar with a SD in the ~1 pN range. Thus, the unstructured linkers lead to the tumbling freedom of the RCK ring, but the tumbling orientation does not matter as *F*_(z)_ and *F*_(xy)_ of the four subunits balance each other independent of the RCK domain orientation.

Next, I considered the work performed by the WLC springs between the bundle crossing and the RCK ring in gating. As work is path dependent, and only closed-pore–RCK-apo and open-pore–RCK-Ca^2+^ structures are available, I assumed the simplest paths that account for the known structures and the gating process (2, 3, 8, 13, 25). Thus, I considered two transition states: a first state, where an activated open conformation RCK ring with widely spread Ser115_(open)_ pulls on closed conformation pore domain Ile99_(closed)_, following the logic that the initial step following Ca^2+^ binding to the RCK ring is a conformational change within RCK ring (Fig. 2 *D*, *Left*), and a second state, where a closed conformation RCK ring with narrow Ser115_(closed)_ is connected to open conformation pore domain Ile99_(open)_, following the logic that upon Ca^2+^ unbinding from the RCK ring, the RCK ring will be first to relax back into the apo conformation (Fig. 2 *E*, *Left*). Alternatively, I consider the first as the conformation that the channel would have to reach when it closes in electrophysiology experiments under saturating Ca^2+^ conditions, while the second would correspond to an opening in the absence of Ca^2+^ (*Discussion*). Under the assumption that the linkers are always unfolded (other than in the solved closed conformation), I calculated all linker lengths, *L*, and the vertical, *L*_(z)_, and radial, *L*_(xy),_ components of these intermediates (Fig. 2 *D* and *E*, *Middle*) and derived the corresponding WLC-spring pulling forces *F* = 22.4 ± 4.7 pN, *F*_(z)_ = 3.4 ± 3.5 pN, and *F*_(xy)_ = 15.5 ± 2.7 pN for the first (Fig. 2 *D*, *Right*, gray shaded, *n* = 23) and *F* = 7.9 ± 0.4 pN, *F*_(z)_ = 5.7 ± 0.7 pN, and *F*_(xy)_ = 4.2 ± 0.4 pN for the second (Fig. 2 *E*, *Right*, gray shaded, *n* = 23) transition state. Again, averaging the forces for each hypothetical transition structure resulted in sub-pN average force deviations between the structures owing to the geometrical relationship between the four subunits (Fig. 2 *D* and *E*, *Right*, white background, *n* = 6). No structure of such a transition state has been reported, and they would likely be short lived (*Discussion*). Conceptually, one could imagine that time-resolved cryo-EM (26, 27), where MthK channels were frozen right after laser-induced Ca^2+^-uncaging or right after Ca^2+^ withdrawal, could resolve such transition states, assuming a sufficiently rapid freezing process.

Using this simple transition path model, I calculated the energies of work performed during the gating process. The first path of work is described by the loading of the WLC springs from the closed to the transition state, i.e., the movement of Ser115_(closed)_ to Ser115_(open)_ tethered to Ile99_(closed)_ (Fig. 2 *F*, *Left*). The work loading the WLC springs amounts to 3.8 ± 1.5 *k*_B_T (Fig. 2 *F*, *Right*, gray shaded, *n* = 23), and again due to the geometric relationship of the subunits of the tetrameric RCK-ring tumbling below the tetrameric pore domain, the work averaged over each structure is nearly identical 3.8 ± 0.3 *k*_B_T (Fig. 2 *F*, *Right*, white background, *n* = 6). Thus, this hypothetical transition state is a high-energy state and short lived because its conversion to the open state is energetically downhill. Indeed, the second path of work considers unloading the WLC springs with the movement of Ile99_(closed)_ to Ile99_(open)_, opening the bundle crossing, tethered to Ser115_(open)_. This process leads to the open-state conformation (Fig. 2 *G*, *Left*). Opening the bundle crossing above an activated Ca^2+^-bound RCK ring is energetically favorable and 3.3 ± 1.5 *k*_B_T of the WLC-spring potential energy is liberated (Fig. 2 *G*, *Right*, gray shaded, *n* = 23), an average 3.3 ± 0.1 *k*_B_T to reach each of the open conformation structures (Fig. 2 *G*, *Right*, white background, *n* = 6). The third path describes the transition from Ser115_(open)_ to Ser115_(closed)_ tethered to Ile99_(open)_, just after unbinding of Ca^2+^ from the RCK ring and the initial step of closing the channel (Fig. 2 *H*, *Left*). This transition is accompanied by a 1.8 ± 0.4 *k*_B_T lowering of the elastic potential energy leading to a state with rather relaxed linkers. The radial force on the linker is only *F*_(xy)_ = 4.2 ± 0.4 pN (Fig. 2 *E*, *Right*), and given that the lateral movement of Ile99_(open)_ to Ile99_(closed)_ is only 1.1 nm (Fig. 2 *C*, *Right*), the closing of the channel is within thermal noise (1 *k*_B_T = 4.14 pN nm) from this transition. To enter the closed state, a fourth path must involve the movement of Ile99_(open)_ to Ile99_(closed)_ tethered to Ser115_(closed)_ and is discussed in the next paragraph.

### A Notable Outlier.

In one of the six open-state MthK structures (PDB 6u68, Fig. 1 *D*, *Right*, 13), one of the four subunits has a folded linker extending from the open-state pore-lining helix, skewing the RCK ring into a dramatically tilted configuration (Fig. 2 *I*, *Left*). Therefore, the linker of the neighboring subunit is in a highly stretched configuration (Fig. 2 *I*, *Middle*, dashed red line) with length *L*_outlier_ = 36.2 Å generating a force *F*_outlier_ = 20.3 pN much higher than all other subunits (Fig. 2 *I*, *Right*, see also the outlier data point in Fig. 1 *E* and *F*). The other 22 “normal” subunits in all structures have rather consistent length with small standard deviation (SD) *L*_normal_ = 29.1 ± 2.8 Å generating *F*_normal_ = 12.8 ± 2.4 pN (*n* = 22, Fig. 2 *I*, *Right*). Also, the two other subunits in PDB 6u68 (not the folded linker subunit or the outlier subunit) are canonical, with linker lengths *L* = 29.5 Å, *F* = 12.9 pN and *L* = 28.5 Å, *F* = 12.1 pN.

Based on the difference in linker lengths of the outlier subunit *L*_outlier_ = 36.2 Å compared to all other canonical subunits *L*_normal_ = 29.1 Å, I could estimate the excess WLC-spring potential energy stored in the outlier (Eq. **8**); it is 2.7 *k*_B_T (Fig. 2 *I*, *Right*). I suggest that the outlier subunit with its highly stretched linker pays the energetic cost for the folded linker and that the energetic gain of folding the linker should be the same or, more likely, slightly higher than 2.7 *k*_B_T. This energy estimate should be important to evaluate the transition to the closed conformation. In a fourth path, as mentioned above, following the reversion of Ser115_(open)_ to Ser115_(closed)_, the bundle-crossing closes, Ile99_(open)_ to Ile99_(closed)_, and the linkers fold. Indeed, in transition state 2, the radial outward pulling force on the linkers is low, 4.2 pN (Fig. 2 *E*, *Right*), and the radial path to close the bundle-crossing is short, 1.1 nm (Fig. 1 *C*, *Right*), and therefore, the work cost of the fourth path should be of order of *k*_B_T (1 *k*_B_T = 4.114 pN nm). Thus, an energy gain of 2.7 *k*_B_T or higher for folding a linker should drive the channel to close along the fourth path.

## Discussion

In this theoretical work, I analyze one closed and six open conformation MthK structures (13) in the framework of the forces generated by the unfolded polypeptide linkers between the RCK ring and pore domain using the WLC polymer extension physics model (14). The closed structure provided 4 Ile99_(closed)_ and 4 Ser115_(closed)_ coordinates, while the six open structures provided 24 Ile99_(open)_ and Ser115_(open)_ coordinates, defining the starting and end points and thus the lengths of the linkers connecting the RCK domain and the pore domain. Of the 24 open structure subunits, 1 linker in one subunit remained folded, leading to another 1 exceptionally extended linker in another subunit within the same structure (*A Notable Outlier*).

**Fig. 3.**
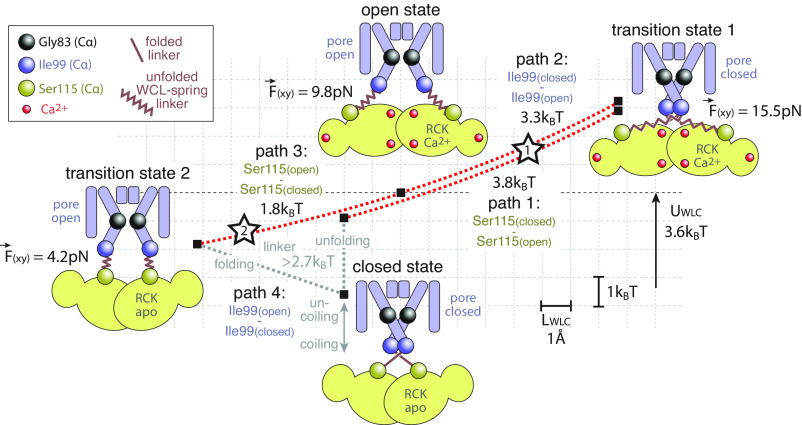
Forces and energetics of bundle-crossing gating in MthK. The closed, transition 1, open, and transition 2 states (black squares and schematics) in an elastic potential energy vs. linker extension length landscape (red dashed lines). Starting from the closed state, upon Ca^2+^ binding (red spheres) to the RCK domain, Ser115 (yellow spheres) move to a radially wider position, unfolding the linker regions (thick red lines) to become entropic springs (red zigzag lines) that pull on Ile99 (blue spheres). 3.8 *k*_B_T work is exerted to load the linker springs (path 1), leading to a hypothetical transition state 1, where a readily activated RCK domain pulls with a radial activating force of 15.5 pN on a still closed pore-domain bundle crossing. Kinking the pore-lining helix at Gly83 (black spheres) allows opening of the bundle-crossing. As Ile99 move outward, 3.3 *k*_B_T of elastic potential energy is released to reach the open state (path 2). The bundle crossing is kept stably open as the linkers exert a radial gating force of 9.8 pN that stabilizes the open conformation. Thus, under Ca^2+^-bound conditions, the open state is a low-energy state (compare open state and transition state 1). Upon Ca^2+^ unbinding, Ser115 move back to a radially narrow position releasing another 1.8 *k*_B_T of elastic potential energy in the linkers (path 3), leading to a hypothetical transition state 2, where the linkers are rather relaxed and the radial force of 4.2 pN provides only little bias on the bundle crossing. Closing the cycle, Ile99 move back to their closed-state position with linker refolding (path 4). Based on the outlier subunit analysis, an energy gain >2.7 *k*_B_T is associated with linker folding (gray dashed lines). Likely linker four-helix coiling provides additional energy to drive closing of the channel in path 4 (gray double-headed arrow). Thus, in Ca^2+^-free conditions, the closed state is a low-energy state (compare closed state and transition state 2). During transition path 1 while Ser115_(closed)_ moves outward to Ser115_(open)_, Ile99_(closed)_ might readily move outward to Ile99_(open)_, thus leading to a relaxation of the linker WLC spring before (at open star 1) full extension is reached (at transition state 1). Transition state 1 should be visited during closed gate transitions in electrophysiology experiments in abundance of Ca^2+^. During transition path 3 while Ser115_(open)_ moves inward to Ser115_(closed)_, Ile99_(open)_ may readily move inward to Ile99_(closed)_, and the linker fold before (at open star 2) full linker relaxation is reached (at transition state 2). Transition state 2 should be visited during open gate transitions in electrophysiology experiments in the absence of Ca^2+^. The dashed lines of path 1 and path 2 (path 3) are slightly vertically offset for clarity of illustration only. The red dashed lines represent the elastic potential energy vs. *L*_WLC_ extension graph of one individual linker only (scale bars indicated).

Summarizing, based on the MthK cryo-EM structures and the WLC model, I propose the following physical scenario of MthK bundle-crossing gating in the framework of a WLC-spring linker extension vs. energy landscape (Fig. 3). The schematic displays the potential energy in an individual unfolded WLC spring linker in the open and transition states (Fig. 3, red dashed lines) and the lateral forces, *F*(xy) that it exerts on the pore domain (Fig. 2 *C*–*H*). The closed state has a folded linker of which I could estimate the energy (Fig. 3, gray dashed lines) from the outlier linker analysis (Fig. 2*I*).

Starting from a closed-pore–RCK-apo MthK, upon Ca^2+^ binding to the RCK domain, the gating ring undergoes a conformational change leading to a spreading of Ser115_(closed)_ to Ser115_(open)_. Ca^2+^ binding to the RCK ring is cooperative with a high Hill coefficient and a *K*_D_ of ~1 mM (9, 10, 25, 28), and there are a total of 6 Ca^2+^-ions bound to the RCK ring of an open-state MthK subunit (13). Based on this affinity, I estimate an upper limit of free energy, ~40 *k*_B_T (6.9 *k*_B_T per ion, ΔG^0^ = −RT ln*K*_Eq_), that Ca^2+^ binding to the RCK domain could provide to gate the channel. I suggest that the displacement of Ser115 immediately leads to the unfolding of the linker (Fig. 3, gray dashed line, linker unfolding), and thus, the linker becomes an entropic spring that gets loaded as Ser115_(closed)_ moved outward to reach the Ser115_(open)_ location exerting a radial pulling force, 15.5 pN on the pore domain, to prime the channel for opening. The work needed to reach this first transition state 1 (a hybrid structure where the readily activated RCK domain with Ser115_(open)_ is connected to a still closed pore domain with Ile99_(closed)_) is 3.8 *k*_B_T (Fig. 3, path 1). Given the strong radial pull, 15.5 pN, the bundle-crossing gate should rapidly open by a movement of Ile99_(closed)_ to Ile99_(open)_. This movement relaxes the extension of the linker spring, as Ile99_(open)_ moves closer to Ser115_(open)_, and the potential energy in the linker decreases by 3.3 *k*_B_T (Fig. 3, path 2). In the open conformation (corresponding to the experimentally determined structure), the linker exerts 9.8 pN radial gating pull keeping the bundle-crossing open. Upon Ca^2+^-unbinding from the RCK domains, Ser115_(open)_ reverts to Ser115_(closed)_, reaching a transition state 2 (a hybrid structure where Ser115_(closed)_ of an apo RCK domain is connected to a still open pore-domain Ile99_(open)_). During this transition, the linkers relax further, releasing another 1.8 *k*_B_T elastic potential energy (Fig. 3, path 3). In this state, each linker only exerts a radial force of 4.2 pN on Ile99_(open)_, and since the radial inward motion in the next step (Fig. 3, path 4) to close the bundle crossing, i.e., Ile99_(open)_ to Ile99_(closed)_, is only ~1.1 nm, the associated work on each linker should be of order of *k*_B_T (1 *k*_B_T = 4.114 pN nm). From the outlier analysis (Fig. 2*I* and *A Notable Outlier*), I estimate that the fold of a linker alone amounts for an energy gain of >2.7 *k*_B_T (Fig. 3, gray dashed line, linker folding), but the coiling of the four pore-lining helices of the four subunits in the closed conformation, with linker residues 99 to 105 further extending these helices, is likely providing further energy to the folded linker state and should further help driving the channel from transition state 2 to the closed state (Fig. 3, gray arrow, linker coiling). Unfortunately, I could not derive an estimate of how much energy the formation of the four-helix bundle adds in the full channel setting. The mechanical polymer extension model used here directly connects the free-energy difference between the states and the work performed during their transition (the heat dissipation is accounted for in the entropy change of the polymer).

But is this scenario realistic? There are supporting arguments for such a physical scheme. First, MthK gating has been extensively studied using electrophysiology (2, 11, 12): In saturating Ca^2+^ (and ideal conditions), the MthK open probability, *P*_O_, is close to unity, with reported *P*_O_ of 0.95 (28), *P*_O_ of 0.96 (10), and *P*_O_ of 0.994 (12). These *P*_O_ values correspond to Δ*G* of −2.9 *k*_B_T (28), −3.2 *k*_B_T (10), and −5.1 *k*_B_T (12) (note that these equilibrium free-energy values of this favorable *P*_O_ have a negative sign). In addition, some of the fast closings are due to rapid block by Ca^2+^ ions in the selectivity filter rather than from bundle-crossing gating conformational changes (10), and thus, the open probability at the bundle-crossing gate is certainly higher than the experiments suggest (2, 29, 30), and therefore, the Δ*G* < −5.1 *k*_B_T. The closure of the channel at saturating Ca^2+^ relates to the movement of Ile99_(open)_ to Ile99_(closed)_ tethered to Ser115_(open)_ (Fig. 3, path 2) that the WLC model characterizes with *U*_path 2_ = 3.3 *k*_B_T per subunit, giving 13.2 *k*_B_T for four subunits.

In the absence of Ca^2+^ (presence of ethylenediaminetetraacetic acid (EDTA)), the MthK open probability, *P*_O_, is essentially zero, with reported *P*_O_ of 0.0001 (28), *P*_O_ of 0.0003 (10), and *P*_O_ of 0.0001 (11). These *P*_O_ values correspond to Δ*G* of 9.2 *k*_B_T (28), 8.1 *k*_B_T (10), and 9.2 *k*_B_T (11). The opening of the channel in the absence of Ca^2+^ relates to the movement of Ile99_(closed)_ to Ile99_(open)_ tethered to Ser115_(closed)_ (Fig. 3, path 4) that implicates the unfolding of the linker that the physical model estimates at *U*_path 4_ > 2.7 *k*_B_T per linker based on the excess elastic potential energy load on the outlier subunit (Fig. 2*I* and *A Notable Outlier*), giving >10.8 *k*_B_T for four subunits. As mentioned above, I consider that the uncoiling of the four helices provides additional energy that is not reported in the outlier subunit analysis in this process. Of course, there are limits to the precision afforded by electrophysiological approaches, and *P*_O_s very close to 0 and 1 require monitoring single molecules for hours to distinguish whether an open or closed state occurred with a frequency of 1 in 10,000 vs. 1 in 100,000. Regardless, the functional measurements clearly suggest that several *k*_B_T are needed to transfer the closed-pore–RCK-apo MthK to an open-pore (>9.2 *k*_B_T), and the open-pore–RCK-Ca^2+^ MthK to adopt a closed pore (< −5.1 *k*_B_T), in good correspondence with the physical analysis of the linkers.

Second, interesting observations have been made regarding the structure and function of the pore domain: In an extensive analysis of MthK channel gating, a paradoxical observation was made: At pH >8.1, some rare channels were active or even constitutively open in the absence of Ca^2+^. These authors provided evidence that these channels had damaged/disassembled RCK rings. Thus, without a meaningful RCK ring interface assisting the folding and tetramer-coiling of the linker domains, the pore domain “alone” may remain open (28). In addition, the MthK pore-domain structure, following proteolytic cleavage (“limited trypsin digestion”) of the linker region and RCK ring (31), revealed pore-lining helices in the open conformation, suggesting that without linker folding, four-helix-coiling, and linker folding onto the apo-RCK domain surface, the open-state pore domain is a low-energy state (31). Interestingly, this 1.45 Å X-ray structure after limited trypsin digestion ended also precisely at Ile99 (31), like the full-length cryo-EM open-state structures (13). Thus, without linkers on which the RCK ring pulls or which contribute energy through folding and coiling that would stabilize the closed state, the open-pore state is quite favorable.

Third, it has been reported that increased temperature facilitated Ca^2+^-dependent opening and increased the open probability of MthK (25, 32). Clearly, increased temperature would favor unfolding of the linker and it to become a WLC spring; also, increased temperature should, according to Eq. **7**, increase *F*_(WLC)_ and thus promote the open state. Therefore, temperature dependence of MthK activation and activity (25, 32) should be well integrated in the framework of this work. In this context, it would be interesting to experimentally test the influence of chaotropic agents on MthK function, as they might promote linker unfolding and favor or lead to channel opening through WLC-spring pulling on the pore domain at lower Ca^2+^ concentration or even in its absence.

Are the proposed transition states realistic? As depicted in Fig. 3, when the channel changes from the closed to the open state, it is not necessary that the linkers get stretched all the way to reach transition state 1 and then relax back to the open state. It is conceivable that at intermediate linker stretching as Ser115_(closed)_ moves to Ser115_(open)_ (e.g., at open star 1 in Fig. 3), the bundle crossing readily opens by the movement of Ile99_(closed)_ to Ile99_(open)_, and the linker spring starts shortening to the open state. On the other hand, in electrophysiology experiments under saturating Ca^2+^ conditions where the RCK ring likely never loses its Ca^2+^ ions and remains in the activated state, transition state 1 should be visited during the rare closings. This is in agreement with the high-energy barrier to leave the open-pore state in electrophysiology experiments in optimized activating conditions where the *P*_O_ is close to 1. For transition state 2, the situation is similar. Perhaps the linkers readily start to fold, Ile99_(open)_ moves to the Ile99_(closed)_ coordinates, while the RCK ring moves back to its apo conformation, Ser115_(open)_ to Ser115_(closed)_ and the linkers are still relaxing (e.g., at open star 2 in Fig. 3). Yet, it seems reasonable that transition state 2 is the conformation that is adopted by the channel during the rare openings in electrophysiology experiments under Ca^2+^-free (EDTA-buffered) conditions. Again, the high-energy barrier to reach that state as proposed in the physical model is in good agreement with the *P*_O_ of almost 0 in such conditions. Currently, we lack information about such possible intermediates, but time-resolved cryo-EM (26, 27), single-molecule Förster (fluorescence) resonance energy transfer (33), and high-speed AFM height spectroscopy (34) might be able to record structures and/or signals of the existence of such short-lived intermediates.

Previously, it has been proposed that the linker in the BK channel, the eukaryotic homologue of MthK, acted as a passive spring in channel gating (35). In that work, the authors shortened and lengthened the linker, and—as one would expect for mechanical coupling—the shorter linker increased the coupling and thus the open probability in the pore upon Ca^2+^ activation, while channels with longer linkers had a decreased open probability upon Ca^2+^ activation. In addition, the authors showed that in the absence of Ca^2+^, the shortened linkers favored channel opening by voltage as compared to the wild-type length linker, while more voltage was needed to activate channels with an elongated linker. In the absence of structural data, the authors estimated a spring constant between 7.7 pN/nm and 18 pN/nm for the four linkers acting in parallel. The more recently solved BK-channel structures revealed that the linkers were extended peptide stretches without secondary structural elements, but they were ordered and structurally resolved (36, 37). Thus, I could not apply the WLC model to estimate the forces and elastic potential energy that they exerted. However, I could measure the real BK linker length and extend the line of thought developed by Niu et al. (35) and calculate a spring constant following their rationale, it would be 12.3 pN/nm and 15.1 pN/nm for the apo and Ca^2+^-activated states, respectively (for the four linkers in parallel). While the WLC model does not provide one spring constant, i.e., the force increases nonlinearly with extension (Figs. 1*D* and 2*I*), I could derive the typical spring constant at the MthK mean linker length, *L*_normal_; it would be 8.1 pN/nm (Eq. **10**). Thus, it appears that a similar mechanism is preserved between BK and MthK channels: A structurally resolved (without secondary structural elements) linker spring in BK channels and an entropic spring constituted of an unfolded linker in MthK translate the Ca^2+^-binding-induced conformational change in the RCK ring into a pulling force to gate the bundle-crossing gate. Similar experiments as formerly performed for BK, changing the linker lengths, could be performed with MthK (given that such channels could be expressed). According to the WLC model, in MthK, a −3-residues shortened or +3-residues lengthened linker would generate forces of ~20 pN and ~9 pN, respectively, with potential energies of 5 *k*_B_T and 2.8 *k*_B_T, respectively (where the wild type generates 12.8 pN and has a potential energy of 3.6 *k*_B_T), if the geometrical constraints between RCK domain and pore domain would remain roughly the same.

Here, I calculated, using the well-established WLC polymer extension physical model (14), the forces and energies involved in MthK-channel gating (Fig. 3). I think that the functional mode of MthK, namely the fact that the pore domain is gated by means of unfolded linkers that are pulled by the RCK ring (and the fact that many open conformation structures are available reporting about 23 linker configurations), serendipitously provided an objective way to access the equilibrium physical parameters of bundle-crossing gating. Given that all cation channels share the same helix–pore-loop–helix pore-domain architecture, I propose that similar forces and energies should be at play to gate the pore domain from the variety of sensory domains that induce gating in other channels.

## Methods

### Structural Analysis: Linker Lengths.

First, I aligned all seven MthK structures (Fig. 1 *A* and *D*) on the parts that are constant between all structures, namely the pore domain starting at the beginning of transmembrane helix 1 until the glycine-hinge in transmembrane helix 2, i.e., residues 20 to 83. To simplify the following calculations and visualization, all structures were also aligned in a way that the fourfold pore axis of the closed structure and the pore domains of all open structures coincided with the z-coordinate axis (Fig. 2 *A* and *B*). Next, I calculated the lengths of the unfolded linkers in the open conformation structures (Figs. 1*D* and 2 *C*, *Left*), L_(WLC)open_, following Eq. **1** (subsequently used to calculate the force that the entropic WLC springs generate, using Eq. **7**):

[1] $$\begin{array}{c} L_{(WLC)open}=\sqrt{{\left(X_{Ser115,N,O}\;-\;X_{Ile99,N,O}\right)}^{2}+{\left(Y_{Ser115,N,O}\;-\;Y_{Ile99,N,O}\right)}^{2}+{\left(Z_{Ser115,N,O}\;-\;Z_{Ile99,N,O}\right)}^{2}}. \end{array}$$

To separate the total lengths of the unfolded linkers into their vertical and radial components, respectively, I assess the linker lengths following Eqs. **2** and **3**. Note that all structures were initially oriented with the fourfold and pore axis coinciding with the z-coordinate axis, therefore *L*_(z)_ directly relates to the vertical and *L*_(x, y)_ the radial components:


[2] $$\begin{array}{c} L_{(z)open}\;=\;\sqrt{{\left(Z_{Ser115,N,O}\;-\;Z_{Ile99,N,O}\right)}^{2}}, \end{array}$$

[3] $$\begin{array}{c} L_{(xy)open}\;=\;\sqrt{{\left(X_{Ser115,N,O}\;-\;X_{Ile99,N,O}\right)}^{2}+{\left(Y_{Ser115,N,O}\;-\;Y_{Ile99,N,O}\right)}^{2}}. \end{array}$$

In Eqs. **1–3**, *Ser115* and *Ile99* stand for the positions of the Cα atoms of Ser115 and Ile99, respectively, in subunits *N* of open conformation structure *O* (Fig. 2 *C*, *Middle*).

To calculate the lengths of the linkers in hypothetical, and certainly very short-lived, transition states (Fig. 2 *D* and *E*, *Left*), *L*_(WLC)trans_, I assume chimeric structures where the activated or apo RCK domain is connected to a closed or open pore-domain bundle-crossing, respectively, following:


[4] $$\begin{array}{c} L_{(WLC)trans}\;=\;\sqrt{{\left(X_{Ser115,N,(O,C)}\;-\;X_{Ile99,N,(C,O)}\right)}^{2}+{\left(Y_{Ser115,N,M(O,C)}\;-\;Y_{Ile99,N,(C,O)}\right)}^{2}+{\left(Z_{Ser115,N,(O,C)}\;-\;Z_{Ile99,N,(C,O)}\right)}^{2},} \end{array}$$

[5] $$L_{(z)trans}\;=\;\sqrt{{\left(Z_{Ser115,N,(O,C)}\;-\;Z_{Ile99,N,(C,O)}\right)}^{2}},$$

[6] $$\begin{array}{c} L_{(xy)trans}\;=\;\sqrt{{\left(X_{Ser115,N,(O,C)}\;-\;X_{Ile99,N,(C,O)}\right)}^{2}+{\left(Y_{Ser115,N,(O,C)}\;-\;Y_{Ile99,N,(C,O)}\right)}^{2}}. \end{array}$$

where *Ser115* and *Ile99* stand for the positions of the Cα atoms of Ser115 and Ile99, respectively, *N* stands for the subunit in any open, *O*, and closed, *C*, state structure (Eqs. **4–6** assess the linkers of transition state 1 or transition state 2 using coordinates *O* and *C* or *C* and *O*, respectively) (Fig. 2 *D* and *E*, *Middle*). Since the closed structure is fourfold symmetric, and therefore, all coordinates in the closed conformation are identical with respect to the fourfold axis, the use of subunit *N* is unambiguous, and the associated coordinates are identical with respect to the symmetry axis.

From these linker length measurements, the corresponding forces, *F*, and their directional components, *F*_(z)_ and *F*_(xy)_, generated by the linkers are calculated using Eq. **7** (Fig. 2 *C*–*E*, *Right*).

### WLC Model: Forces.

The WLC model is a well-established model that describes the extension of a polymer chain. The Marko-Siggia interpolation formula (Eq. **7**) is generally used in and has been corroborated by protein unfolding experiments to describe the force generated by the extension, *x*, of an unstructured amino acid chain. Force is needed to stretch a polymer chain up to the maximal extension of its backbone length, i.e., the contour length, *L_C_*, that is the multiple of the persistence length, *l_p_*, of each constituent unit, in MthK this is the linker residues between Ile99 and Ser115.


[7] $$\begin{array}{c} F_{\left(WLC\right)}\;=\;\frac{k_{B}T}{l_{p}}\left[\frac{1}{4}(1-x/L_{c}{)}^{-2}+\;x/L_{c}\;-\;\frac{1}{4}\right]. \end{array}$$

The persistence length, *lp*, of amino acid residues in a polypeptide chain has been determined many times by AFM force spectroscopy experiments and is *l_p_* = 0.38 nm (6, 23, 38, 39). The intuition behind the polymer stretching models is the following: A fully stretched peptide chain with end-to-end length ≅ contour length can only adopt close to 1 possible configuration, while a strongly coiled peptide chain with end-to-end length << contour length can adopt many configurations. Thus, the stretched peptide is a low-entropy and high free-energy state, while the coiled peptide is a high-entropy and low free-energy state. Stretching the peptide chain from coiled to extended thus leads to a continuous decrease of possible configurations and an increase in energy. Therefore, work must be done to stretch the peptide along its extension length reaction coordinate, and the peptide acts as an entropic spring, where the force, *F*_WLC_, increases nonlinearly with extension (Fig. 1*D*). In the case of the MthK linker, the mean open-state WLC spring is *L*_(WLC)_ = 29.4 ± 3.1 Å (Fig. 1*E*); the longest linker is 36 Å (*A Notable Outlier*). Following Eq. **7**, the mean force generated by the open-state linkers is *F*_(WLC)_ = 13.1 ± 2.8 pN (Fig. 1*F*).

To evaluate the force that acts on the bundle-crossing gate to pull the bundle-crossing open or maintain the open conformation, I separated the force applied by the WLC spring into a parallelogram of forces with two normal components, a vertical force, *F*_(z)_ in the direction of the fourfold axis and a lateral force pulling radially outward from the fourfold axis, *F*_(xy)_. The force components *F*_(z)_ and *F*_(xy)_ of each individual linker (WLC spring) are determined from the vertical and radial positions of the linker endpoints and the respective lengths of the linkers in vertical *L*_(z)_ and radial *L*_(xy)_ directions (Eqs. **2**, **3**, **5**, and **6**). In addition, I calculated for each structure the average of the total force, *F*_-struct_, and its components *F*_(z)-struct_ and *F*_(xy) -struct_ through averaging the forces exerted by its linkers (Fig. 2 *C*–*E*, white background). This showed that the average forces in all structures were very similar with sub-pN SDs. It cannot be excluded that slight asymmetries exist in the individual domains of the structures, especially during the transitions. However, the tumbling RCK domain represents certainly by far the largest asymmetry, but the generated forces average out. This is due to the geometrical relationship of the 4 subunits, where—due to the tumbling of the RCK domain with fixed tetrameric structure—one RCK subunit that is located close to the membrane plane, small *F*_(z)_, and radially wide outward, large *F*_(xy)_, has a diagonally opposite located RCK subunit that is located far from to the membrane plane, large *F*_(z)_, and close to the fourfold axis, small *F*_(xy)_, (Fig. 2*B*). This geometric relationship between the subunit locations of the tumbling tetrameric RCK ring leads to linker WLC-spring forces that are very similar between all structures.

### WLC Model: Energy.

To evaluate the potential energy in and the work associated with loading and unloading the WLC springs during channel gating, I integrated the Marko–Siggia interpolation formula (Eq. **7**):


[8] $$U_{\left(L\right)}^{0}\;=-\int_{L_{1}}^{L_{2}}F_{\left(WLC\right)}\cdot dx,$$

to generate the relationship:


[9] $$\begin{array}{l} U_{\left(L\right)}^{0}=-\frac{k_{B}T\cdot L_{C}}{lp}\\ \;\;\;\;\;\;\;\;\;\;\;\left[\frac{1}{4}\left(\frac{1}{1-L_{2}/L_{C}}-\frac{1}{1-L_{1}/L_{C}}\right)+\left(\frac{L_{2}^{2}}{L_{C}^{2}}-\frac{L_{1}^{2}}{L_{C}^{2}}\right)-\frac{1}{4}\left(\frac{L_{2}}{L_{C}}-\frac{L_{1}}{L_{C}}\right)\right]. \end{array}$$

This integrated WLC interpolation model was used to calculate the work to load and the release of elastic spring potential energy in the linker WLC springs during gating (Fig. 3, path 1, path 2, path 3), as well as to calculate the excess potential energy stored in the outlier subunit with respect to the normal linker lengths (*A Notable Outlier*) to estimate the energy of linker refolding (Fig. 3, path 4).

### WLC Model: Spring Constant.

To estimate spring constants, *k*_(WLC)_, along the WLC profile, the derivative of the Marko–Siggia interpolation formula (Eq. **7**) is taken, resulting in Eq. **10**, which allows to calculate the slope at any linker extension, here used to derive the spring constant of the canonical MthK open-state linker length, *L*_normal_ = 29.1 Å (the average length of the 22 normal canonical linkers in the open-state structures), giving 8.1 pN/nm.

[10] $$\begin{array}{c} k_{\left(WLC\right)}\;=\;\frac{dF_{\left(WLC\right)}}{dx}\;=\;\frac{k_{B}T}{l_{p}}\left[\frac{1}{2L_{c}}{\left(1\;-\;x/L_{c}\right)}^{-3}+\;1/L_{c}\right]. \end{array}$$

## Data Availability

All data and methods are in the manuscript or are available online. All MthK structures analyzed here are available on the PDB website (accession codes: 6u6d, 6u6h, 6uxb, 6uxa, 6u6e, 6ux7, 6u68, solved and deposited by the Nimigean laboratory (13).
